# The Digital Atlas of Ancient Rare Diseases (DAARD) and its relevance for current research

**DOI:** 10.1186/s13023-024-03280-0

**Published:** 2024-07-24

**Authors:** Julia Gresky, Melina Frotscher, Juliane Dorn, Kristina Scheelen-Nováček, Yannick Ahlbrecht, Tina Jakob, Toni Schönbuchner, José Canalejo, Benjamin Ducke, Emmanuele Petiti

**Affiliations:** 1 Division of Natural Sciences, German Archaeological Institute, Berlin, Germany; 2 Department of Archaeology, Durham University, Durham, UK; 3 Cuprit GbR, Leipzig, Germany; 4 Central Research Services/IT, German Archaeological Institute, Berlin, Germany

**Keywords:** Achondroplasia, Stunted growth, Short stature, Database, FAIR principle, Paleopathology, Archaeology, History, Medical collections

## Abstract

**Background:**

The history of rare diseases is largely unknown. Research on this topic has focused on individual cases of prominent (historical) individuals and artistic (e.g., iconographic) representations. Medical collections include large numbers of specimens that exhibit signs of rare diseases, but most of them date to relatively recent periods. However, cases of rare diseases detected in mummies and skeletal remains derived from archaeological excavations have also been recorded. Nevertheless, this direct evidence from historical and archaeological contexts is mainly absent from academic discourse and generally not consulted in medical research on rare diseases.

**Results:**

This *desideratum* is addressed by the Digital Atlas of Ancient Rare Diseases (DAARD: https://daard.dainst.org), which is an open access/open data database and web-based mapping tool that collects evidence of different rare diseases found in skeletons and mummies globally and throughout all historic and prehistoric time periods. This easily searchable database allows queries by diagnosis, the preservation level of human remains, research methodology, place of curation and publications. In this manuscript, the design and functionality of the DAARD are illustrated using examples of achondroplasia and other types of stunted growth.

**Conclusions:**

As an open, collaborative repository for collecting, mapping and querying well-structured medical data on individuals from ancient times, the DAARD opens new avenues of research. Over time, the number of rare diseases will increase through the addition of new cases from varied backgrounds such as museum collections and archaeological excavations. Depending on the research question, phenotypic or genetic information can be retrieved, as well as information on the general occurrence of a rare disease in selected space–time intervals. Furthermore, for individuals diagnosed with a rare disease, this approach can help them to build identity and reveal an aspect of their condition they might not have been aware of. Thus, the DAARD contributes to the understanding of rare diseases from a long-term perspective and adds to the latest medical research.

## Background

### Rare diseases in human archaeological remains

Rare diseases play a crucial but often overlooked role in human history, and human bones from archaeological contexts provide the only direct evidence for these diseases. Paleopathology, the transdisciplinary study of ancient diseases from multiple sources, can contribute to medical knowledge of the long-term genetic, pathophysiological, and social evolution of rare conditions.

Among the oldest examples known thus far are the more than 10,000-year-old case of chondrodystrophic dwarfism from Italy (Romito, Paleolithic) [1] and one individual, the oldest and currently only known individual affected by autosomal-dominant osteopetrosis, from Albania dated to 4600 BCE [2]. However, when and where did rare diseases such as trisomy, osteogenesis imperfecta or melorheostosis first occur?

These questions are difficult to answer because rare diseases are even less common in archaeological human remains than in modern contexts, and reports on rare diseases are often hidden in less accessible journals or mentioned as supplementary information in archaeological publications [3].

Ancient medical texts and iconographic sources cover only parts of human history; they deliver an incomplete geographical picture, and their information is distorted through the lens of old medical knowledge and preconceptions. Thus, human remains are the only direct source, specifically, those from museum collections and archaeological contexts.

Historic collections comprise a large variety of specimens, often from individuals with a rare disease, as these individuals were frequently collected during certain periods [4]. Many of these specimens are cataloged and accessible (e.g., Charité Museum of Medical History Berlin, Germany, or the Pathological-Anatomical Collection in the Narrenturm, Vienna, Austria). However, there are many cases that are either not accessible or have a questionable diagnosis that needs to be re-evaluated, e.g., osteopetrosis in the Fairbank collection [5].

The enormous corpus of skeletons and mummies from archaeological contexts represents an invaluable source of information that remains largely unexplored because better known and more widespread diseases [3] are prioritized over rare conditions. Furthermore, when present, most of the data are hidden in unpublished (gray) literature, making them difficult to access.

### Creating visibility and awareness of ancient rare diseases

Visibility remains the core problem for ancient but also for modern diseases. Prompting a turning-point strategy, the solution presented in this manuscript, the Digital Atlas of Ancient Rare Diseases (DAARD), addresses two main aims. Firstly, by adding new cases of rare diseases, medical specialists can consult data from more individuals. These cases can be tested against recent diagnoses, and due to their different states of preservation, they can offer different research approaches. The second aim is to involve patient associations and the wider public. Creating awareness of the history of rare diseases helps to build identity and adds an important, thus far underrepresented, aspect to the knowledge of rare diseases. To achieve these aims, we created the first database of rare diseases in archaeological human remains, the Digital Atlas of Ancient Rare Diseases.

The DAARD is an open-access GIS-based database that is part of the iDAI world (https://idai.world/) of the German Archaeological Institute (Deutsches Archäologisches Institut; DAI) in Berlin. It is available online at https://daard.dainst.org. The DAARD is a unique multimedia repository in which published data on rare diseases from archeological and museum contexts are collected and organized according to geographical, chronological, biological, and archaeological features. Furthermore, the guided interactive data-entry mask provides a tool to inspect and validate the diagnostic process.

In clinical research, databases on rare diseases have led to great advances in diagnostic methods and they have become very important in helping clinicians in the field of often unknown, diverse syndromes. These databases also create awareness of the variety of possible diseases, since medical staff due to their usual daily routine might not be familiar with these diseases. Such useful diagnostic tools include Phenomizer [6, 7] and FindZebra [8, 9]. These databases aim to provide a full dataset for the phenotypical expression of each rare disease, including skin and other soft tissue involvement and laboratory values. The study of ancient human remains is limited by preservation due to taphonomic conditions, in most cases leaving only teeth and skeletal tissues to evaluate pathological changes, with the exception of mummified human remains. However, mummies are not commonly found, but when they are present, such as in Egypt or South America, they offer a valuable source of information because of preserved soft tissue preservation.

The DAARD is supplementing clinical databases as it concentrates on bone changes in a more detailed way compared to clinical databases. In addition to providing important information on different rare diseases, and their geographical and chronological distributions, this open-access database enables us to construct a broader picture of the pathological and social history of rare diseases.

## Methods

The DAARD is a publicly accessible WebGIS application, designed and hosted by the DAI, and implemented in 2022 by Cuprit GbR. It utilizes GeoNode (https://geonode.org), an open-source content management system for spatial data known for its modular design and extensive integration of diverse spatial data formats.

In turn, GeoNode relies on other established open-source components, such as the following:

1. Django (https://www.djangoproject.com/) is used for system administration tasks (such as user management) and provides a robust authentication and authorization system.
2. Postgres/PostGIS (https://www.postgresql.org/) serves as the database’s backend, offering advanced geospatial capabilities for efficient storage and retrieval of data.
3. GeoServer (https://geoserver.org/) acts as a middleware component, enabling dynamic map rendering and efficient spatial data dissemination via the geodata service provider standards defined by the Open Geospatial Consortium (OGC).

GeoNode’s usefulness is amplified by the support of a dedicated community, which is vital for the continuous evolution and refinement of these components. These key features make it an ideal base for the DAARD.

The DAARD allows for the entry of 18 different rare diseases and currently comprises 127 individuals (last update May 22, 2024). Already published but also new data from prehistoric and historic individuals affected by a rare disease are underpinning research.

The database is organized into five sections, with each of them presenting different data. Entering new cases is facilitated by an intuitive and straightforward process. The first section comprises data about the chosen disease and individual data about the affected human remains. The disease in question can be chosen; however, only rare diseases already represented in the DAARD are available for entry. Age at death (fetus, infans, adolescent, adult, senile, unknown) and biological sex (male, female, indeterminable) can be chosen from dropdown menus. The second section, “Inventory”, queries the completeness of the skeleton and the preservation of individual bones (absent, less than 75% preserved, more than 75% preserved). The skeleton is divided into different sections (e.g., cranial district, axial skeleton, left upper limb). The third section is used to query the pathological “bone changes”. For each bone, specific changes can be chosen from dropdown menus for the respective disease, choosing macroscopic, radiographic, and/or microscopic examination methods. The fourth section details the data entry for the “Site” or origins of the human remains. Here, archaeological context information, such as the place of excavation, funerary context and chronology, is documented, in addition to information about the current place of curation of the human remains. In the fifth and last section “Genetic analyses and publication”, it is possible to indicate whether biomolecular studies such as DNA analyses have already been carried out. This aspect will be improved and given more prominence in future versions of the DAARD. In this last section, any publications of the case are also entered into the database. Throughout the data entry process, users have the flexibility to return to individual sections, allowing for the addition, removal, or correction of entries. In cases where the provided selection criteria in the dropdown menu do not cover the relevant information, users can make free-text entries (e.g., age-freetext). Once all relevant details are completed, the case can be submitted. However, before it will become visible as a new entry on the DAARD world map, it will be reviewed by a member of the scientific board.

## Results

### Presentation of the DAARD using an example of rare diseases involving short stature

In the DAARD cases of rare diseases in mummified and skeletonized human remains are collected, providing as much information as possible on the affected individual and its historical or archaeological context.

When opening the DAARD website, all individuals affected by a rare disease are identified by colored dots on the world map, with each disease represented by a different color. The different colors are detailed in the legend, which provides an immediate overview of the distribution of a specific disease around the world.

**Fig. 1:**
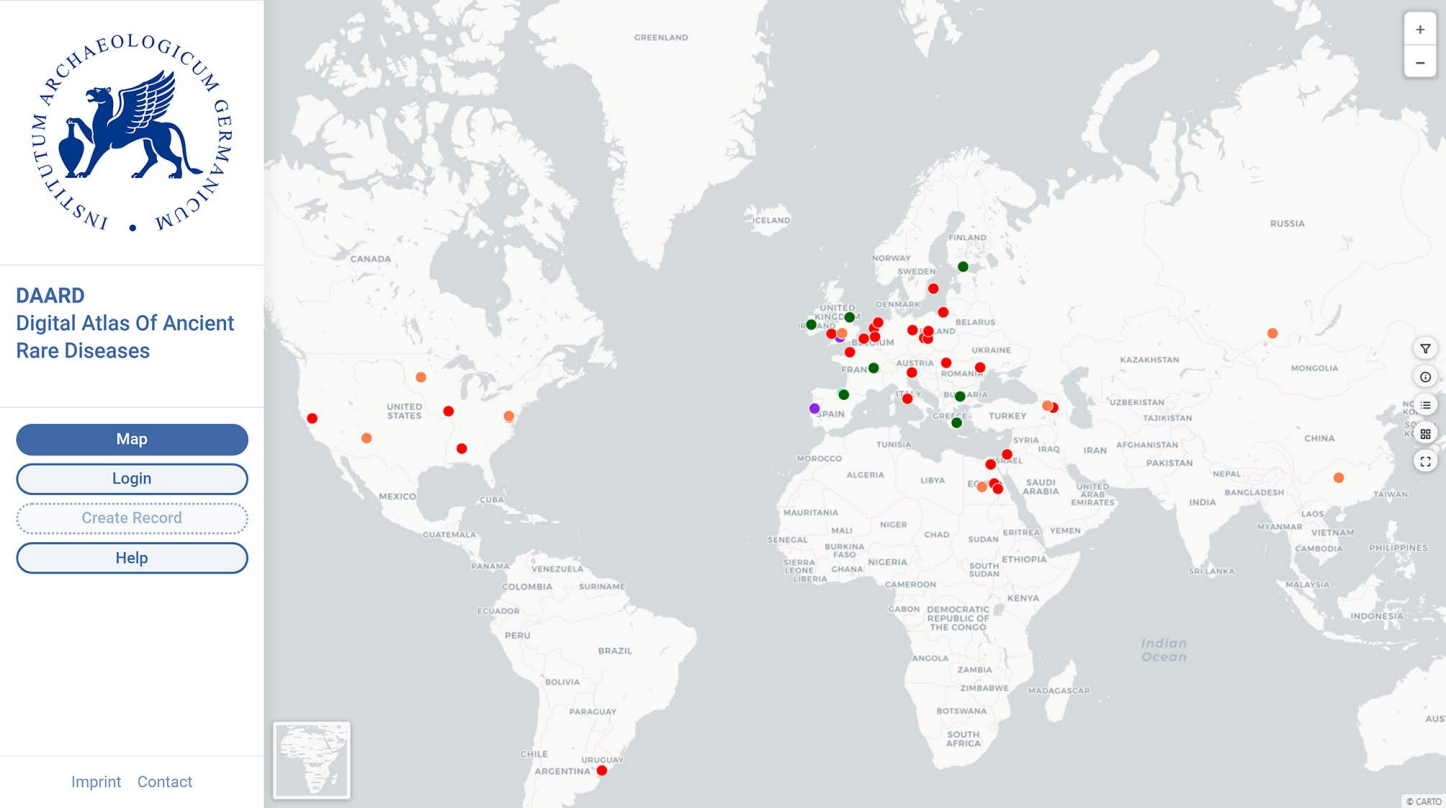
Individuals with short stature recorded in the DAARD (achondroplasia (*n* = 30) in red, hypopituitarism (*n* = 9) in orange, trisomy 21 (*n* = 9) in green, trisomy 18 (*n* = 1) in turquois, and Turner syndrome (*n* = 2) in purple). Some dots represent several individuals which become visible when zooming in

In the paleopathological literature, one of the most frequently recorded physical characteristics, which might be indicative of a rare disease, is short stature [3]. At present, 51 entries for diseases associated with short stature are compiled in the DAARD (Fig. 1) (last update May 9, 2024). The list of diagnoses thus far includes achondroplasia (*n* = 30), trisomy 18 and 21 (*n* = 10), hypopituitarism (*n* = 9), and Turner syndrome (*n* = 2). The oldest of these cases is the already mentioned individual from Romito, Italy [1] who was diagnosed with chondrodystrophic dysplasia. The youngest case of short stature is found in a person who died in Washington, D.C., USA, in 1959 and whose skeleton is curated at the Robert J. Terry Anatomical Collection in the same city. This individual’s short stature is diagnosed as hypopituitarism [10]. The DAARD also includes other diseases that can lead to short stature, e.g., Albright Hereditary Osteodystrophy, but cases of these diseases are not yet been included in the database.

### One of the most frequently found rare diseases: Achondroplasia

**Fig. 2:**
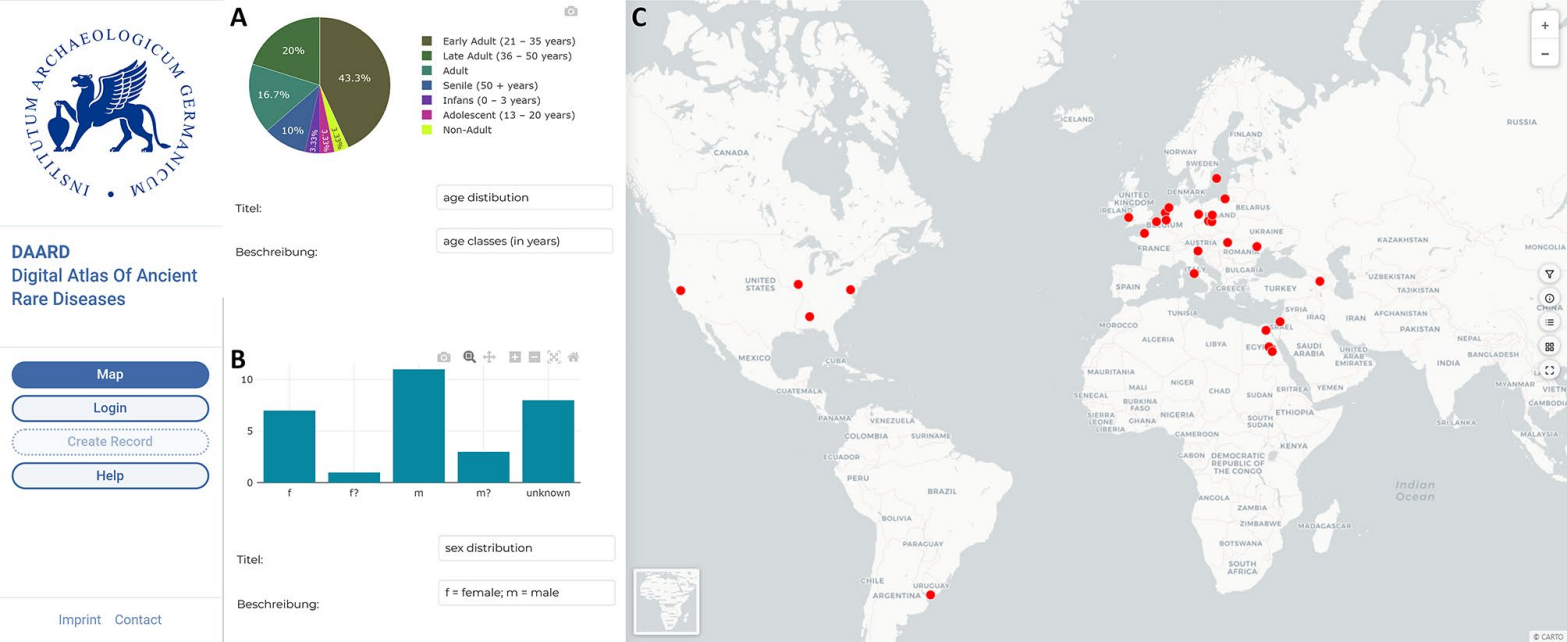
Distribution of **A**) age and **B**) sex among the 30 individuals affected by achondroplasia: recorded in the DAARD, as well as **C**) their geographic location. Some dots represent several individuals which become visible when zooming in

The most frequently reported cause of short stature in archaeological human remains is achondroplasia, for which 30 entries are currently available in the database (Figs. 1 and 2). Targeted queries on various aspects and research questions about individuals affected by achondroplasia can be initiated by using specific filter settings. For example, choosing additional filter criteria enables the researcher to display or hide certain characteristic criteria, such as age groups, a particular biological sex, or entire temporal categories. Furthermore, numerous other attributes can be filtered, such as skeletal characteristics, place of origin (archeological site), or place of curation, e.g., collections in museums or research institutions. The ability to selectively query specific aspects significantly expedites literature research, as the database compiles all registered cases, allowing for direct use of data and easy extraction of basic information about specific diseased individuals from the database attribute table.

Entering data into the designated form facilitates the transfer of data into the defined terminology, ensuring standardization and enabling a refined search for specific terms. This approach is also possible for papers in languages other than English, as their contents can be translated into English and then added to the database.

Most of the achondroplasia cases (Fig. 1) that were identified in the database originated from excavations in Egypt (*n* = 6/30). The second largest group included individuals who were almost equally distributed across Europe (*n* = 16/30). Only eight individuals were found in non-European locations, five were from North America, two from Asia, and one was from South America. However, this does not reflect the actual geographical distribution of the disease; it rather indicates research gaps visible in the underrepresentation of some geographical areas where few or no archaeological projects have been conducted thus far. Therefore, the DAARD may help to identify research gaps and support the selection of new research projects and strategies for researchers worldwide.

### Additional tools of the DAARD

**Fig. 3:**
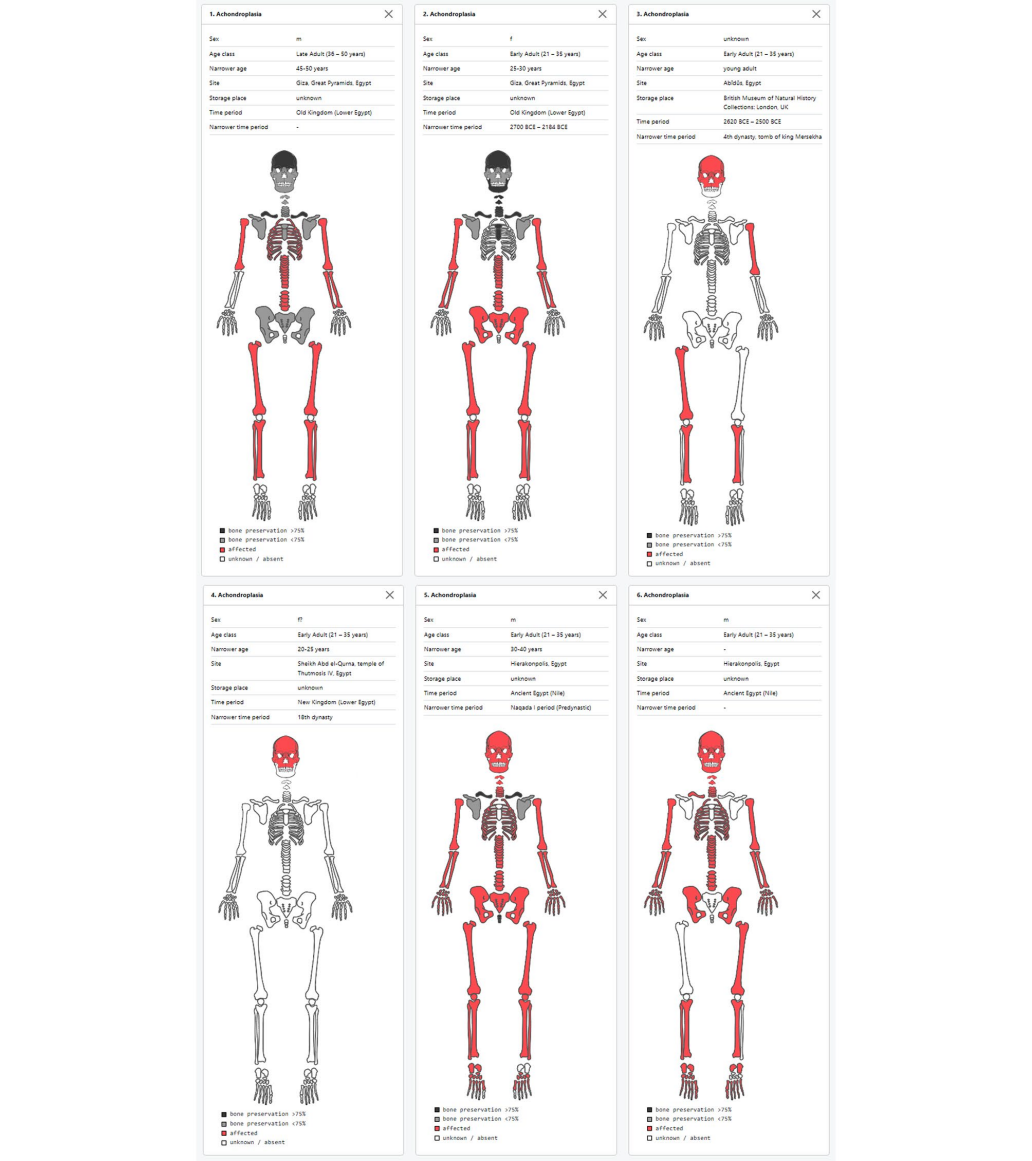
Comparison of bone preservation and affected bones in achondroplastic individuals from Egypt: recorded in the DAARD

The DAARD offers several tools to support geographically and historically wide-ranging research questions, such as comparative views of tables, graphs (Fig. 2) and skeletal diagrams. For the latter, the available bones are schematically colored in different shades of gray (light gray: < 75% preserved, dark gray: > 75% preserved), and affected bones are indicated in red (Fig. 3). Thus, data on preservation of several skeletons can be compiled to provide an immediate overview of post-depositional biases, distinguishing skeletal areas that are simply not preserved from those that are actually not affected (Fig. 3). This tool is particularly useful when dealing with diseases that can affect different parts of the skeleton, allowing for the targeted selection of individuals of interest. The compilation of skeletal diagrams can be downloaded directly from the DAARD and used as comparative illustrations for publication (Fig. 3).

The morphological changes associated with each disease are stored in the database’s backend and can be chosen when entering a new case. This enables a search and comparison of the data. For example, it is possible to search for certain cases of this disease as well as for certain morphological traits, such as a depressed nasal bridge or other typical features of achondroplasia.

When comparing the six Egyptian individuals in the DAARD, it became evident that only four individuals exhibited macroscopic features of macrocephaly (Fig. 3). In the two Old Kingdom individuals, cranial involvement was notably absent, which is rather atypical for the clinical manifestation of achondroplasia. Conversely, limb involvement is consistently observed in all individuals where limbs are present. A comprehensive understanding of the distinctions and commonalities among individuals is achievable only through a meticulous comparison of cases within the DAARD. Therefore, the DAARD serves as a crucial foundation and source of information for subsequent research endeavors.

Specific research methodologies might provide additional information of interest to scientists. For each case, it is indicated whether only macroscopic methods have been applied or whether additional radiographic, microscopic, isotopic, or genetic analyses have been undertaken. References for corresponding publications are given. So far, genetic analysis was performed for only one of the 30 individuals with achondroplasia. The historic, 180-year-old skeleton of an achondroplastic adult male is currently curated at the Museum Vrolik, the Anatomical Museum of the University of Amsterdam, The Netherlands. Boer and colleagues [11] were able to extract nuclear DNA from a premolar and identified the Gly380Arg mutation of the FGFR3 gene, which is pathognomonic for achondroplasia.

All known publications for each individual are deposited in the DAARD because, in some cases, several re-evaluations have taken place, and the original data are distributed across several publications. Information about preserved skeletal remains is valuable for researchers who want to identify skeletons for further evaluation. When possible, this information was extracted from the publications; in some cases, the authors were contacted, and their additional information was added. Of the 30 individuals with achondroplasia, six were curated in museums, and four were housed in collections of universities or institutes. For 20 individuals, no information about the location of the skeletal remains could be obtained, but any new information about their place of curation will be added to the database.

## Discussion

### Evidence for rare diseases in the literature and iconography

There is interest in the history of rare diseases, evidenced by a number of books and papers, particularly on people with short stature, and specifically on achondroplasia. The book “Small People - Great Art” [12] focusses on individuals of short stature in popular paintings. A large corpus of iconographic evidence exists for Ancient Egypt [13, 14] and pre-Columbian South America [15, 16]. However, iconographic evidence can be highly subjective, for the artist and the observer. 2D visualization makes a thorough and accurate diagnosis very difficult, and no original material from the affected person is available for further diagnosis.

### Archaeological human remains (skeletons, mummies) as objective sources

Human skeletal remains and mummies from archaeological excavations are mainly kept in the storage facilities of local archaeological institutions, universities, or museums. Historical human remains are often part of anatomical collections in universities and museums [4, 17]. These institutions often grant access to bona fide researchers on request. Despite their sometimes less than ideal preservation, they “can yield valuable contributions in e.g., etiopathogenetic issues and potentially expand the literature beyond the restrictions of single case studies due to the plurality of specimens with similar anomalies in these collections” ( [11] page 6). However, often, from today’s perspective, the provenance of such human remains from historical collections may be ethically questionable. To follow ethical guidelines the acquisition history of each individual should be clarified and individuals should not be included in future research projects if found to be acquired by unethical means [18].

Furthermore, in contrast to iconographic evidence of rare diseases, human remains as bioarcheological sources provide objective information, whose presence is not the result of intentional selection due to economic, social or political reasons or unintentional due to historical and cultural filters as it is the case for iconographic evidence.

### Problems in diagnosing rare diseases in archaeological human remains

The diagnosis of rare diseases in human remains from prehistoric or historic archaeological contexts or osteological collections is not easy, and comparative cases are difficult to find in the literature. Diagnosing this highly diverse disease class is often challenging, as the resulting bone changes are commonly not part of the basic paleopathological education program, and most specialists are not familiar with them [19]. The diagnosis of rare diseases in ancient skeletal human remains is further complicated by the fact that formerly existing soft tissue changes are no longer observable, and no reference data from clinical and laboratory analyses are available. On the one hand, the low visibility of rare diseases results from a combination of missed diagnoses or misdiagnoses [3]. On the other hand, visibility is strongly dependent on the type of publication, such as preferred article types and journals. Human skeletal remains and mummies from individuals who were affected by a rare disease are almost exclusively published as case reports; these reports are currently not overly popular in science [20] and they are less often read and cited compared to larger-scale group or population-based studies. It is therefore difficult to publish such studies in scientific journals, which makes them more likely to be hidden in the gray literature [3].Until now, these limitations have prevented the development of a comprehensive review of the pathological and social history of rare diseases.

### The DAARD as a repository of information on ancient rare diseases, applying FAIR (findable, accessible, interoperable, reusable) principles for data management

**Fig. 4:**
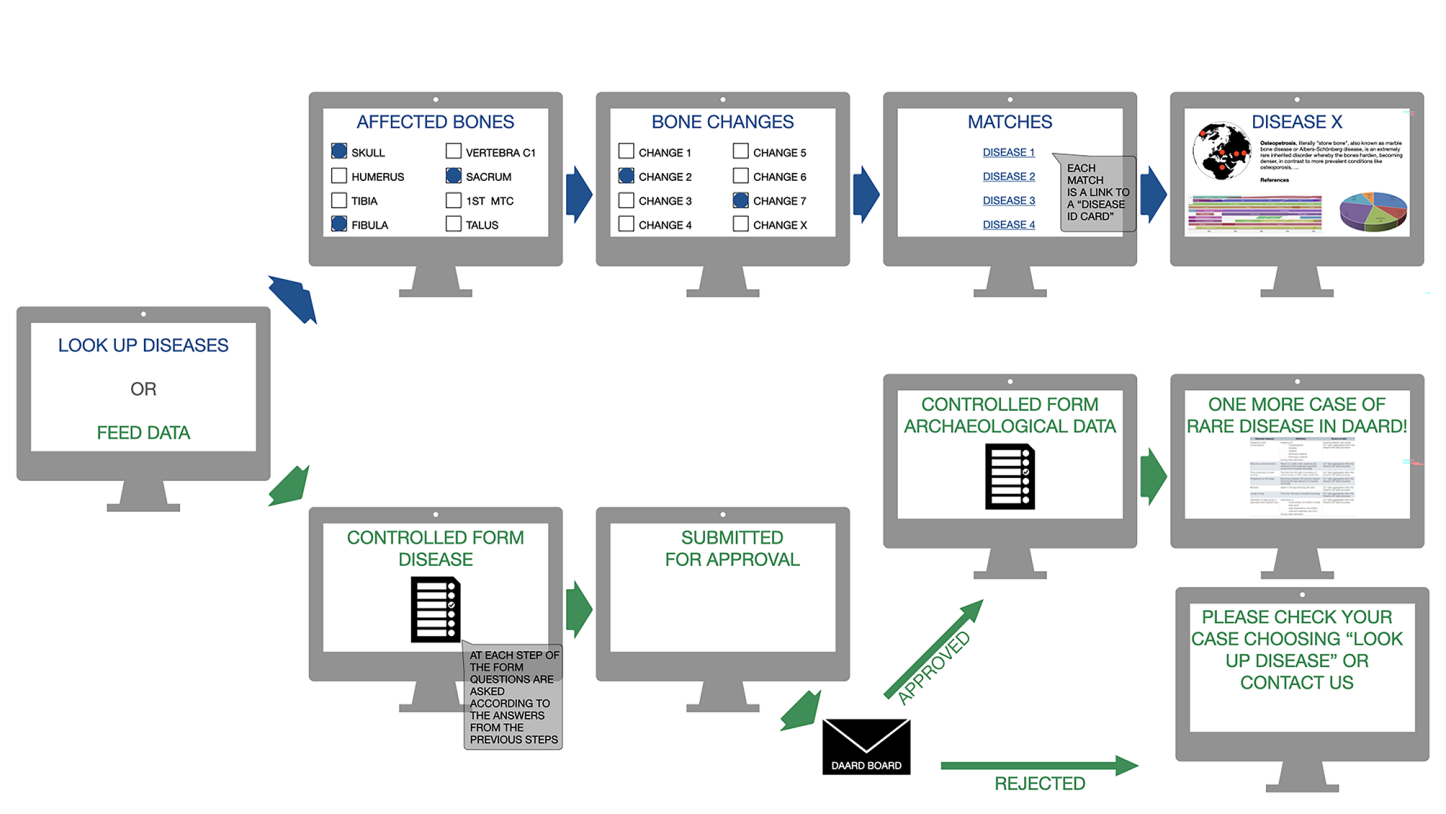
Summary of the workflow of the DAARD, illustrating its two main functions: data search and retrieval, and data feeding

The DAARD is an open access platform for collecting new or already published cases of rare diseases, serving as a repository (Fig. 4). Given that not all publications are open access, entering their basic data into the DAARD makes them accessible to everyone, thus promoting the FAIR principle of data presentation [21]. The DAARD refers to the original paper and its DOI for further information. Newly identified cases can be directly entered by the respective researcher, and published disease cases can be added by any user. This approach maximizes the amount of data input and helps to further develop the database.

All data entries undergo a review process before they appear in the list and as dots on the world map. The review process is conducted by a team of physicians and biological anthropologists, and the data has to be approved before their final upload.

### Limitations of the DAARD

Ultimately, the DAARD is reliant on the interest of researchers to include their data. Furthermore, only diseases that are already documented in the DAARD system can be included and of the large number of rare diseases affecting the skeleton, only a small fraction has been recorded in the DAARD. Currently, there is only a skeletal schematic drawing of an adult skeleton, representing both adult and non-adult skeletons. In the future skeletal drawings will be adapted to the different age classes of the affected individuals. With the increasing importance of genetic analysis in the diagnosis of rare diseases more emphasis will be put on genetic evidence as a vital part of the DAARD.

Furthermore, the study of ancient rare diseases is severely limited by several factors, ranging from biased burial customs and differences in bone preservation to difficulties in the diagnosis of diseases in skeletal human remains, and the type of publication [3].

### Further applications of the DAARD: medical and social objectives

During the development of the DAARD, we followed two main objectives—one medical and one social: Ancient skeletons, depending on their preservation, can contribute to recent medical research in different ways.

### Medical objectives

Archaeological human skeletal remains can be sampled for further genetic research on their specific disease and this would also verify the initial diagnosis. The probability of finding the same genetic mutation in ancient and recent disease cases is high especially if the disease is pathognomonic, e.g., achondroplasia [11]. However, this assumption cannot be regarded as certain and must be tested using a larger reference sample. The genetic mutations underlying trisomy 21 are found in individuals from the Neolithic period in Ireland [22], Bronze Age Spain, Bulgaria and Greece [23], the Iron Age in Great Britain [24], early medieval France [23, 25] and post-medieval Finland [23]. This mutation is the same as today [26]. Regarding the verification of the possible presence of trisomy in morphologically suspected cases from archaeological contexts, genetic analysis is recommended since the phenotypic expression is highly variable and the diagnosis is difficult on a macroscopic level alone [27]. Less clear is the genetic composition of rare diseases with multiple possible mutations resulting in a similar phenotype, such as autosomal-dominant osteopetrosis (ADO) of which 13 different genetic variants are known. The most common mutation is a heterozygous mutation in the ClCN7 gene [28]. Currently, a genetic investigation of the oldest known case of an individual affected by ADO [2] is being performed to determine the underlying type of genetic mutation.

Despite the challenges in extracting high-quality DNA from ancient tissues and ethical issues of destructive sampling, new methods such as next generation sequencing (NGS) of whole genomes/exomes enables large scale investigations of genetic samples. This would allow for a confirmatory genetic testing of potential rare diseases whenever DNA is available. Information about monogenic conditions, as well as valuable insights into the evolution of genome organization, genetic risk factors and variant distribution are more likely to be achieved by sequencing.

By including affected archaeological human remains, the small number of individuals (recent and ancient) with rare diseases increases, and cautious and well-targeted sampling of analytical material can provide more but also different samples than can be taken from a living patient. By doing so, new datasets for studying rare disorders can be acquired, which will generate knowledge about these very specific diseases throughout humankind. However, neither human remains from historical collections nor archaeological excavations should be exploited to obtain samples, as the “irreplaceable value that these collections have as cultural-historical objects” must not be ignored ( [11] page 7).

### Social objectives

The social aspect focusses on the affected people and their patient association groups that refers to a yet underexplored aspect of rare diseases. Understanding our history is an inherent aspect of human nature. This is an important topic, ranging from family structures to the histories of a population or a social-political group. People affected by a rare disease thus far lack a history of their specific disease. Establishing this historical context is important for building identity and revealing that living with these diseases has been a challenge faced by individuals not only in the present but also by people hundreds or even thousands of years ago. Adding this historical perspective to the disease might be interesting to the affected people and could help them to contextualize their disease, and create more general awareness.

### Future aims

In future, more rare diseases should be added to the DAARD. The better known the database will be, the more cases of individuals with rare diseases will be recognized and can be entered into the database, and a wider picture of each rare disease will emerge. Having generated this database, we hope that it is valuable to the scientific community researching rare diseases as well as for the groups of affected people to add a historic aspect to their diseases. Furthermore, in addition to search and retrieval functions, the increasing number of cases prompts predictive functions that facilitate and standardize differential diagnoses. Provided with a set of pathological changes of skeletal tissues, the DAARD will be able to generate a list of diseases organized on the basis of their most likely association with the observed lesion(s). The accuracy of this process is expected to increase with the number of cases fed into the DAARD.

## Conclusions

With the development of the Digital Atlas of Ancient Rare Diseases (DAARD), the first search tool for ancient rare diseases provides open access to original data and allows for comparisons of characteristics of recent and ancient diseased individuals based on the FAIR principles. This digital atlas allows medical specialists to search for cases of rare diseases worldwide within all time periods and allows affected people to explore the history and even prehistory of their specific rare disease.

## Data Availability

All data generated or analyzed during this study are included in this published article.

## References

[1] Frayer DW, Macchiarelli R, Mussi M. A case of Chondrodystrophic Dwarfism in the Italian late Upper Paleolithic. Am J Phys Anthropol. 1988;75(4):549–65. 3291617 10.1002/ajpa.1330750412

[2] Gresky J, Sokiranski R, Witzmann F, Petiti E. The oldest case of osteopetrosis in a human skeleton: exploring the history of Rare diseases. Lancet DE. 2020;8:3. 10.1016/S2213-8587(20)30307-7 32946815

[3] Gresky J, Dorn J, Teßmann B, Petiti E. How rare is rare? A literature survey of the last 45 years of paleopathological research on ancient rare diseases. Int J Paleopathol. 2021;33:94–102. 33813348 10.1016/j.ijpp.2021.03.003

[4] Berezina N, Buzhilova A. Rare cases of Rare Diseases: re-examining early 20th century cases of Anencephaly from the Collection of the Moscow State University, Russia. Int J Paleopathol. 2021;34:12–9. 34098226 10.1016/j.ijpp.2021.05.008

[5] Horan FT, Beighton PH. Osteopetrosis in the Fairbank Collection. J Bone Joint Surg. 1978;60(1):53–5. 10.1302/0301-620X.60B1.342533 342533

[6] Köhler S, Schulz MH, Krawitz P, Bauer S, Dölken S, Ott CE, Mundlos C, Horn D, Mundlos S, Robinson PN. Clinical Diagnostics in Human Genetics with Semantic Similarity Searches in Ontologies. AJHG. 2009;85(4):457–64. 19800049 10.1016/j.ajhg.2009.09.003 PMC2756558

[7] Köhler S, Vasilevsky NA, Engelstad M, Foster E, McMurry J, Aymé S, Baynam G, et al. The human phenotype ontology in 2017. Nucleic Acids Res. 2017;45(D1):D865–76. 27899602 10.1093/nar/gkw1039 PMC5210535

[8] Dragusin R, Petcu P, Lioma Ch, Larsen B, Jørgensen HL, Cox I, Hansen LK, Ingwersen P, Winther O. Specialised tools are needed when searching the web for rare disease diagnoses. Rare Dis. 2013;1:e2500. 10.4161/rdis.25001 PMC3932942 25002998

[9] Dragusin R, Petcu P, Lioma Ch, Larsen B, Jørgensen HL, Cox I, Hansen LK, Ingwersen P, Winther O. FindZebra: a search engine for Rare diseases. IJMI. 2013;82(6):528–38. 10.1016/j.ijmedinf.2013.01.005 23462700

[10] Kimock C. Descriptions of Dwarfism in the skeletons of two individuals from the Robert J. Terry Anatomical Skeletal Collection. University Honors Capstone. Washington, D.C.: American University; 2013.

[11] Boer L, Naue J, de Rooy L, Oostra R-J. Detection of G1138A mutation of the FGFR3 gene in tooth material from a 180-Year-old museological Achondroplastic Skeleton. Genes. 2017;8:214. 28850094 10.3390/genes8090214 PMC5615348

[12] Enderle A, Meyerhöfer D, Unverfehrt G. Small people - great Art/Restricted growth from an artistic and medical viewpoint. Hamm: Artcolor; 1994.

[13] Dasen V. Dwarfism in Egypt and classical antiquity: Iconography and Medical History. Med Hist. 1988;32(3):253–76. 3063904 10.1017/S0025727300048237 PMC1139882

[14] Kozma Ch. Dwarfs in ancient Egypt. Am J Med Genet. 2006;140A(4):303–11. 10.1002/ajmg.a.31068 16380966

[15] Bacon WJ. The Dwarf Motif in Classic Maya Monumental Iconography: A Spatial Analysis. PhD thesis. University of Pennsylvania, Department of Anthropology; 2007.

[16] Rodríguez CA, Isaza C, Pachajoa H. Achondroplasia among ancient populations of Mesoamerica and South America: Iconographic and Archaeological evidence. Colomb Med. 2012;43(3):212–5. 10.25100/cm.v43i3.1158 PMC4001954 24893194

[17] Oostra R-J, Baljet B, Dijkstra PF, Hennekam RCM. Congenital anomalies in the Teratological Collection of Museum Vrolik in Amsterdam, the Netherlands. I: syndromes with multiple congenital anomalies. Am J Med Genet. 1998;77(2):100–15. 9605284 10.1002/(SICI)1096-8628(19980501)77:2<100::AID-AJMG3>3.0.CO;2-W

[18] Fründt S, Schiffels S, Winkelmann A. Ways of analyzing human remains and the benefits for scientific research. editor. Guidelines. Care for human remains in museums and collections. Berlin: German Museum Association; 2021. pp. 75–84. German Museum Association.

[19] Gresky J. Fehlbildungen und seltene Krankheiten. In: Weber J, Wahl J, Zink A, editors. Osteologische Paläopathologie. Ein Handbuch für Anthropologen, Mediziner Und Archäologen. Berlin: Lehmanns; 2022. pp. 461–88.

[20] Boutin AT, Longo CM, Lehnhard R. The role of case studies in recent paleopathological literature: an argument for continuing relevance. Int J Paleopathol. 2022;38:45–54. 35810660 10.1016/j.ijpp.2022.06.002

[21] https://www.publisso.de/en/research-data-management/fair-principles (accessed January 26, 2024).

[22] Cassidy LM, Maoldúin RO, Kador T, Lynch A, Jones C, Woodman PC, Murphy E, et al. A Dynastic Elite in Monumental Neolithic Society. Nature. 2020;582(7812):384–88. 32555485 10.1038/s41586-020-2378-6 PMC7116870

[23] Rohrlach AB, Rivollat M, de-Miguel-Ibáñez P, Moilanen U, Liira A-M, Teixeira JC, Roca-Rada X, et al. Cases of Trisomy 21 and Trisomy 18 among historic and prehistoric individuals discovered from ancient DNA. Nat Commun. 2024;15:1294. 38378781 10.1038/s41467-024-45438-1 PMC10879165

[24] Anastasiadou K, Silva M, Booth T, Speidel L, Audsley T, Barrington C, Buckberry J, et al. Detection of chromosomal aneuploidy in ancient genomes. Commun Biol. 2024;7(1):14. 38212558 10.1038/s42003-023-05642-z PMC10784527

[25] Rivollat M, Castex D, Hauret L, Tiller A-M. Ancient down syndrome: an osteological case from Saint-Jean-des-Vignes, northeastern France, from the 5–6th century AD. Int J Paleopathol. 2014;7:8–14. 29539495 10.1016/j.ijpp.2014.05.004

[26] Gresky J. Assessing autosomal Aneuploidy in Ancient genomes. TiG. 2024. S0168952524000805. 10.1016/j.tig.2024.04.006 38664113

[27] Halle U, Hähn C, Krause S, Krause-Kyora B, Nothnagel M, Drichel D, Wahl J. Die Unsichtbaren. Menschen Mit Trisomie 21 in Archäologie Und Anthropologie. Arch Inf. 2019;42:1–17.

[28] Kornak U, Kasper D, Bösl MR, Kaiser E, Schweizer M, Schulz A, Friedrich W, Delling G, Jentsch TJ. Loss of the ClC-7 chloride channel leads to osteopetrosis in mice and man. Cell. 2001;104:205–15. 11207362 10.1016/S0092-8674(01)00206-9

